# Does *Bt* rice pose risks to non‐target arthropods? Results of a meta‐analysis in China

**DOI:** 10.1111/pbi.12698

**Published:** 2017-02-20

**Authors:** Cong Dang, Zengbin Lu, Long Wang, Xuefei Chang, Fang Wang, Hongwei Yao, Yufa Peng, David Stanley, Gongyin Ye

**Affiliations:** ^1^ State Key Laboratory of Rice Biology & Key Laboratory of Agricultural Entomology of Ministry of Agriculture Institute of Insect Sciences Zhejiang University Hangzhou China; ^2^ Institute of Plant Protection Shandong Academy of Agricultural Sciences Jinan China; ^3^ State Key Laboratory for Biology of Plant Diseases and Insect Pests, Institute of Plant Protection Chinese Academy of Agricultural Sciences Beijing China; ^4^ Biological Control of Insects Research Laboratory USDA/Agricultural Research Service Columbia MO USA

**Keywords:** *Bt* rice, meta‐analysis, non‐target, functional guilds

## Abstract

Transgenic *Bt* rice expressing the insecticidal proteins derived from *Bacillus thuringiensis* Berliner (*Bt*) has been developed since 1989. Their ecological risks towards non‐target organisms have been investigated; however, these studies were conducted individually, yielding uncertainty regarding potential agroecological risks associated with large‐scale deployment of *Bt* rice lines. Here, we developed a meta‐analysis of the existing literature to synthesize current knowledge of the impacts of *Bt* rice on functional arthropod guilds, including herbivores, predators, parasitoids and detritivores in laboratory and field studies. Laboratory results indicate *Bt* rice did not influence survival rate and developmental duration of herbivores, although exposure to *Bt* rice led to reduced egg laying, which correctly predicted their reduced abundance in *Bt* rice agroecosystems. Similarly, consuming prey exposed to *Bt* protein did not influence survival, development or fecundity of predators, indicating constant abundances of predators in *Bt* rice fields. Compared to control agroecosystems, parasitoid populations decreased slightly in *Bt* rice cropping systems, while detritivores increased. We draw two inferences. One, laboratory studies of *Bt* rice showing effects on ecological functional groups are mainly either consistent with or more conservative than results of field studies, and two, *Bt* rice will pose negligible risks to the non‐target functional guilds in future large‐scale *Bt* rice agroecosystems in China.

## Introduction

Genetically modified (GM) crops expressing *cry* genes derived from *Bacillus thuringiensis* Berliner (*Bt*) have been grown commercially since 1996 to control target insect pests worldwide (Cohen *et al*., 2008). GM crops production have been increasing, amounting to 179.7 million ha over 28 countries in 2015 (James, 2015). Despite the substantial economic and environmental benefits of deploying *Bt* crops (Klumper and Qaim, 2014; Raymond Park *et al*., 2011), real concerns about their ecological risks continue (Brookes and Barfoot, 2015). These concerns drive contemporary ecological risk assessments designed to guide current and future risk management.

Meta‐analyses have been applied widely in ecological risk assessments of *Bt* crops on non‐target arthropods (Comas *et al*., 2014; Naranjo, 2009; Peterson *et al*., 2011). Marvier *et al*. (2007) conducted a meta‐analysis to assess the ecological risks of *Bt* maize and *Bt* cotton on non‐target invertebrates, and reported that certain non‐target taxa were less abundant in *Bt* cotton and *Bt* maize fields compared with insecticide‐free control fields. Naranjo (2009) concluded that negative effects of *Bt* crops on predators and parasitoids in laboratory tests coincided with lower abundance in fields except for *Bt* rice and *Bt* eggplant cropping systems. They detected no differences in the abundances of non‐target organisms in *Bt* rice fields, while another study revealed reduced spider abundances in *Bt* rice cropping systems relative to controls (Peterson *et al*., 2011). These results highlight the need for a comprehensive analysis of the current state of our understanding of ecological risks associated with deploying *Bt* rice lines on a large scale.

Rice, *Oryza sativa* L.*,* is one of the world's most important food crops (Zeigler and Barclay, 2008). Since development of the first transgenic *Bt* rice plant in 1989 (Yang *et al*., 1989), a series of *Bt* rice lines, expressing Cry1A, Cry1C, Cry2A or Cry1Ab/Vip3H, had been generated in China (Chen *et al*., 2011; Li *et al*., 2016). These lines effectively suppress stem borer, leaf folder and other lepidopteran pests (Chen *et al*., 2010; Wang *et al*., 2016; Ye *et al*., 2001a,b). Their environmental risks towards non‐target arthropods have been fully studied in laboratory and field settings (Chen *et al*., 2011; Cohen *et al*., 2008; Li *et al*., 2016). However, the results varied with transgenic rice lines and/or non‐target taxa, and accurate predictions of the influence of *Bt* rice on rice agroecosystems remain problematic. In this report, we addressed this issue by developing and presenting an analysis of the accessible literature on the influence of *Bt* rice on non‐target arthropod ecological functional guilds from laboratory to field conditions in China.

## Results

**Figure 1:**
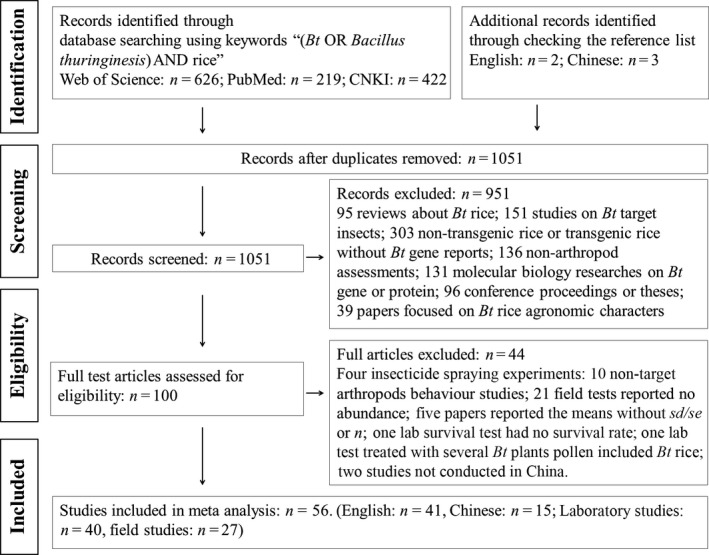
PRISMA flow diagram showing the procedure used for selection of studies for meta‐analysis.

Our database in this analysis contained 282 observations from 40 papers reporting laboratory studies and 585 observations from 27 papers reporting field studies (Tables S1 and S2). Details of the literature search are shown in Figure 1. Most of the data had no publication bias in our database (Figures S1–S3).

### Laboratory studies

**Figure 2:**
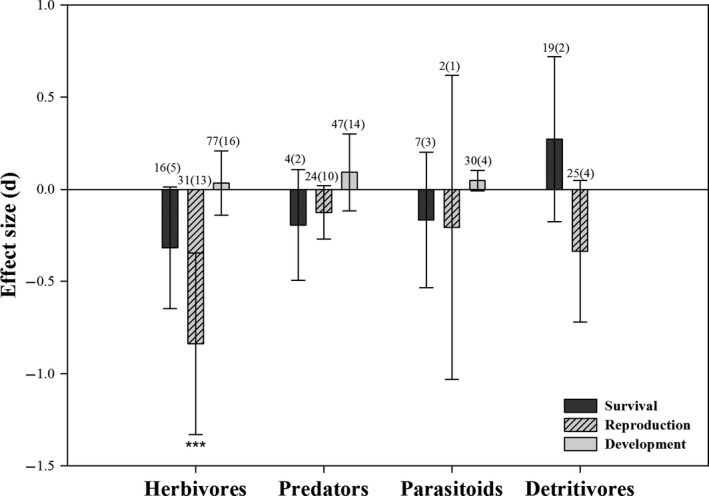
Meta‐analysis of laboratory studies examining the influence of *Bt* rice on non‐target ecological functional guilds biological parameters. For the development of detritivores, no data could be collected to conduct the analysis. Effect size (*E*) is Hedges’*d*, and error bars represent bias‐corrected 95% *CI* (confidence interval). Values above each bar indicate the total number of studies for each group (number of papers). Asterisks denote significant differences in the observed effect sizes among the comparisons (*P <* *0.05; **P <* *0.01; ***P <* *0.001).

Among the three laboratory parameters we assessed, the survival or development of the indicated ecological functional groups were not significantly affected by *Bt* rice (Figure 2; Table 1). Also, the reproduction of predators, parasitoids and detritivores were similar between *Bt* and control rice. The effect size of herbivore reproduction was lower following *Bt* treatments, indicating herbivores on *Bt* rice laid fewer eggs (Table 1), although the herbivore data including thrips and aphids were highly heterogeneous (*I* ^2^ = 93.7%, *P*_heterogeneity_ < 0.001). We re‐analysed the data after removing the information on thrips and aphids, which, again, revealed a significant reduction in herbivore reproduction (*E *=* *−0.449, *P *<* *0.001, *I* ^2^ = 35.8%, *P*_heterogeneity_ = 0.050; Figure S4). We conducted a subgroup analysis to understand the high heterogeneity of the development data on predators. The developmental rates of predators was restrained after feeding on lepidopteran prey from *Bt* rice (*E *=* *1.754, *P *<* *0.001; Figure S5), while other subgroups did not significantly affect by *Bt* rice.

**Table 1:** Influence of *Bt* rice on survival, development and reproduction of the ecological functional groups in laboratory

Functional guilds	Survival		Reproduction		Development	
	*E* (95% *CI*)	*P*	*E* (95% *CI*)	*P*	*E* (95% *CI*)	*P*
Herbivores	−0.318 (−0.648 to 0.012)	0.059	−0.838 (−1.331 to 0.345)	<0.001	0.034 (−0.141 to 0.208)	0.707
Parasitoids	−0.166 (−0.534 to 0.202)	0.376	−0.207 (−1.031 to 0.617)	0.622	0.047 (−0.009 to 0.102)	0.098
Predators	−0.194 (−0.494 to 0.106)	0.205	−0.125 (−0.270 to 0.019)	0.089	0.092 (−0.117 to 0.301)	0.386
Detritivores	0.272 (−0.175 to 0.719)	0.233	−0.336 (−0.719 to 0.048)	0.086	–	–

*E *= effect sizes, *P *= significance level, *CI* = confidence interval. ‘–’ means that no data could be collected to conduct the analysis for the development of detritivores.

### Field studies

**Figure 3:**
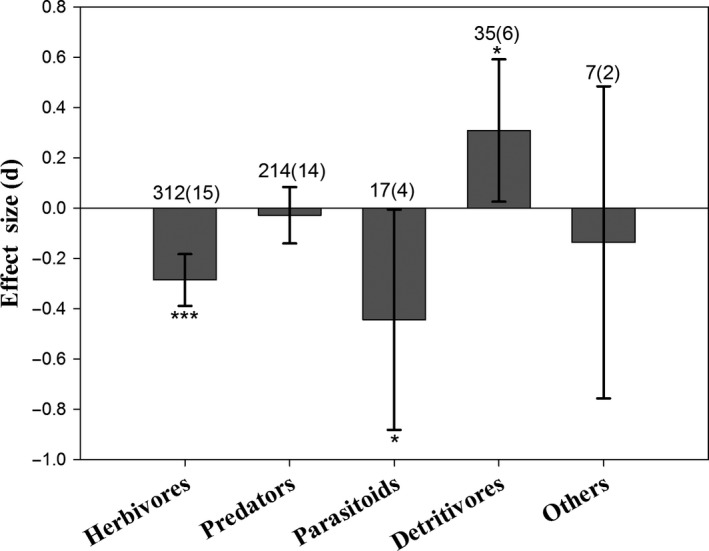
Meta‐analysis of field studies examining the influence of *Bt* rice on non‐target ecological functional guilds abundances. Asterisks denote significant differences in the observed effect sizes among the comparisons (*P <* *0.05; **P <* *0.01; ***P <* *0.001).

**Figure 4:**
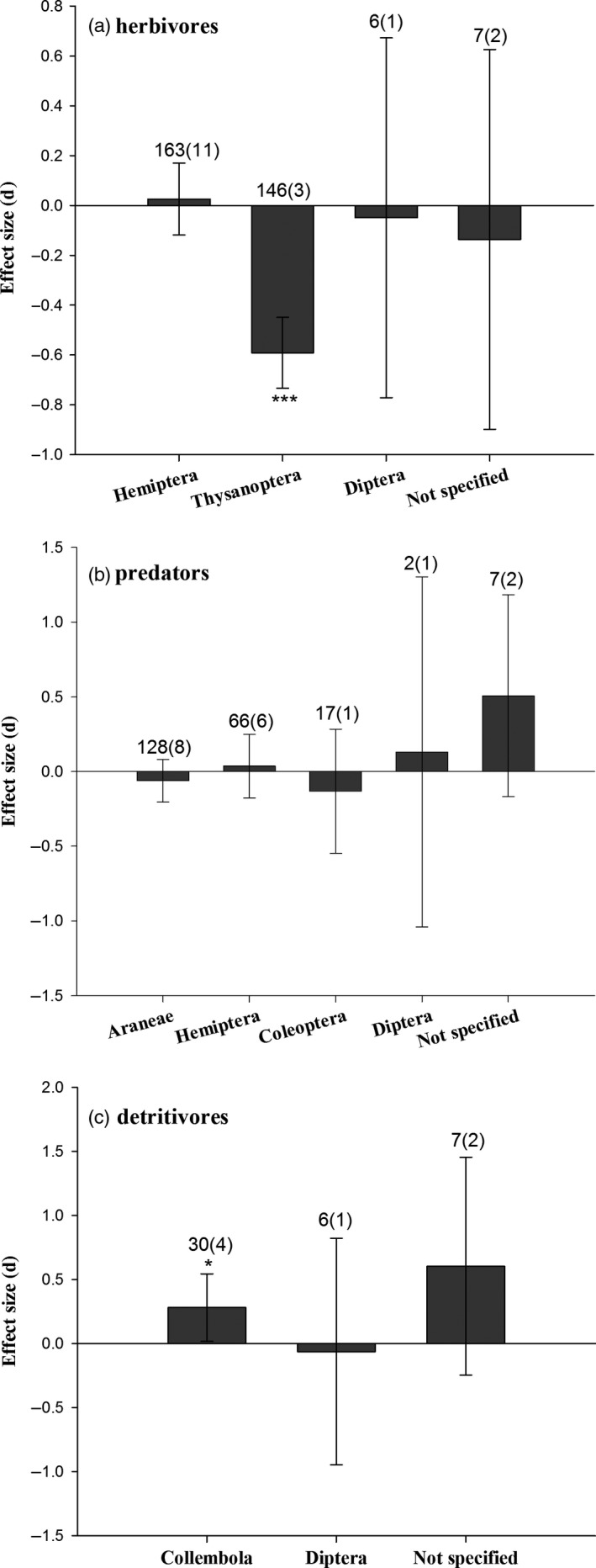
Meta‐analyses of field studies examining the influence of *Bt* rice on the taxa abundance of herbivores (a), predators (b) and detritivores (c). Asterisks denote significant differences in the observed effect sizes among the comparisons (*P <* *0.05; **P <* *0.01; ***P <* *0.001).

The abundance of non‐target herbivores (*E *=* *−0.286, 95% *CI* = −0.389 to −0.182, *P *<* *0.001; Figure 3), including plant‐feeding thrips on *Bt* rice (*E *=* *−0.591, *P *<* *0.001; Figure 4a), was significantly reduced, compared to controls. Predator abundances on *Bt* rice did not differ from controls (*E *=* *−0.028, 95% *CI* = −0.140 to 0.085, *P *=* *0.629; Figure 3). The densities of three main predatory orders, Araneae, Hemiptera and Coleoptera, were similar between *Bt* rice and the control (Figure 4b). There was a slight reduction for the abundance of parasitoids in *Bt* rice paddies (*E *=* *−0.444, 95% *CI* = −0.882 to −0.005, *P *=* *0.048). Populations of detritivores increased in *Bt* rice fields relative to controls (*E *=* *0.309, 95% *CI* = 0.026 to 0.592, *P *=* *0.032), possibly due to higher abundance of Collembola (*E *=* *0.280, *P *=* *0.016; Figure 4c).

## Discussion

Many arthropods provide numerous ecosystem services in rice agricultural systems, such as biological control, pollination, crop residue decomposition and soil health improvement (Hao *et al*., 1998; Wolfenbarger *et al*., 2008). They are classified into ecological functional groups, herbivores, predators, parasitoids and detritivores, based on ecosystem services. Herbivores serve as prey for predators and hosts for parasitoids (Norris and Kogan, 2005). Predators and parasitoids are important natural enemies of crop pests in agroecosystems (Naranjo, 2005), and detritivores contribute to degrading plant litter and microorganisms (Rusek, 1998). Thus, our meta‐analysis focused on impacts of *Bt* rice on these ecological functional guilds. We interpret the overarching results to show that the results of laboratory studies are reasonable predictors of the outcomes of field studies.

The fecundity and field abundances of herbivores were significantly suppressed after consuming *Bt* rice. Thrips and aphids did not account for these differences, because removing the thrips and aphids data did not change the outcome of our analysis. This is reasonable because several studies reported that aphids preferred *Bt* maize or *Bt* cotton compared to controls (Faria *et al*., 2007; Liu *et al*., 2005). *Bt* maize and *Bt* cotton did not impact thrips (Li *et al*., 2007; Obrist *et al*., 2005), although they could accumulate Cry protein from *Bt* cotton (Kumar *et al*., 2014). Other studies also reported that the brown planthopper, a non‐target sucking pest of *Bt* rice, laid fewer eggs on *Bt* rice lines (KMD1 and KMD2) expressing the Cry1Ab protein in laboratory and field surveys (Chen *et al*., 2007, 2012; Gao *et al*., 2011). The results of an additional meta‐analysis on planthoppers were consistent with these reports (Figure S6). Our analyses predicate that populations of the non‐target herbivores including thrips and planthoppers in *Bt* rice fields are no more likely to achieve outbreak levels than insects in non‐*Bt* rice cropping systems. We speculate that thrips and planthoppers laid fewer eggs on *Bt* rice because of the direct actions of *Bt* insecticidal proteins and the indirect impacts of alterations in nutritional qualities of *Bt* rice plants.

Two recent meta‐analyses reported that predators developed more slowly in tri‐trophic tests involving *Bt* crop–herbivore–predator systems (Duan *et al*., 2010; Naranjo, 2009). This pattern may be attributed to the varied types of prey consumed by predators. After feeding on Cry1Ab‐containing rice leafrollers, *Cnaphalocrocis medinalis*, a *Bt*‐targeted pest, the *Bt* protein was present in its predator, the wolf spider, *Pirata subpiraticus*. However, there was neither a binding protein in the brush border membrane vesicles nor an accumulation with longer feeding time for Cry1Ab in the spider. The authors inferred the spiders suffered elongated development times, possibly due to reduced prey quality (Chen *et al*., 2009). Romeis *et al*. (2006) similarly concluded adverse *Bt* effects on predators were due to the reduced prey quality. Given that there are many prey types in rice fields, we infer that *Bt* rice does not influence predator abundance in field studies, in agreement with previous studies (Chen *et al*., 2009; Han *et al*., 2011; Lu *et al*., 2016; Tian *et al*., 2010).

*Bt* treatments led to small reductions in braconid parasitoid abundances in field, but not laboratory studies (Lu *et al*., 2014, 2016; Tian *et al*., 2008). Similarly, *Bt* maize led to negative effects on the parasitoid *Macrocentrus cingulum* Brischke (Hymenoptera: Braconidae) in previous meta‐analyses (Duan *et al*., 2010; Wolfenbarger *et al*., 2008). These findings indicate that the biological performance of some parasitoid species may be adversely affected by *Bt* crops after parasitizing *Bt*‐targeted lepidopteran pests. In our analysis, parasitoids were mainly composed of two families, larval parasitoids, Braconids, which parasitize *Bt*‐targeted pests and egg parasitoids, Mymaridae, which parasitize non‐targeted pests, such as planthoppers. The pooled effect size was significantly lower in *Bt* rice fields. The limited number of studies and the different sampling methods may help understand the variance among studies. Our assessment is based on 17 observations from four papers that met our selection criteria (Bai *et al*., 2012; Lu *et al*., 2014, 2016; Tian *et al*., 2008). This small data set may lead to inaccurate assessments of the effect size. Our interpretation is that combining collection methods in the field, specifically, vacuum suction and sticky cards, may lead to a more complete appreciation of parasitoid populations in future.

We found that the number of surviving springtails, juveniles and adults, did not decrease after feeding on *Bt* rice plant materials or artificial diets containing *Bt* protein in the laboratory. Contrarily, the number of springtails in *Bt* rice cropping systems significantly increased. This effect differed from results with other transgenic crops, because *Bt* maize and *Bt* cotton did not impact detritivore abundance (Wolfenbarger *et al*., 2008). A higher abundance of Collembola was collected in *Bt* rice crops using a vacuum suction method, with no difference in pitfall trap surveys (Lu *et al*., 2014). We infer the ecological services due to detritivores, decomposing and recycling plant residue, would not be affected by *Bt* protein in rice agroecosystems.

In total, our results indicated the *Bt* rice effects on the functional groups obtained from laboratory were not always consistent with that in field trials for the complicated factors existed in paddy agroecosystem. A tiered approach is indispensable to assess the ecological risks of transgenic *Bt* rice in future (Romeis *et al*., 2008). There is no doubt that China is playing a leading role in the development and risk assessment of *Bt* rice (Li *et al*., 2014), and biosafety certificates for commercial planting of two *Bt* rice lines (Huahui 1 and *Bt* Shanyou 63) in Hubei Province have been issued twice by the government in 2009 and 2014 (Li *et al*., 2016). Quantitative syntheses of the risk assessments of *Bt* rice would provide a strong evidence for the Chinese policy makers to avoid some disputes caused by German government in 2009 (Marvier, 2011; Ricroch *et al*., 2010). Despite that the effects of *Bt* rice in China might be not similar with them in other countries because of the ecology of insects/plants and climate zones varied around the world, it would offer some lessons to the country in which *Bt* rice is in urgent need to be developed.

Moreover, although we tried our best to collect data and conduct the analysis, there were still some limitations. Firstly, we failed to make some comparisons between the treatments of *Bt* insecticide protein and chemical insecticide for few cases with chemical insecticide. Also, the data of some functional guilds (especially parasitoids and detritivores) seemed to be not enough. With the knowledge accumulated of risk assessment, these aspects will be gradually improved in future.

## Experimental procedures

### Data search and database production

The database was created by searching the Web of Science (http://isiknowledge.com), PubMed (http://www.ncbi.nlm.nih.gov/pubmed) and China National Knowledge Infrastructure (CNKI, http://www.cnki.net) using the key words ‘(Bt OR *Bacillus thuringinesis*) AND rice’. The following criteria were applied to screen the studies: (i) transgenic rice expresses one or more Cry proteins and targets lepidopteran pests; (ii) non‐transgenic rice was used as control plants in laboratory and field research; (iii) one or more non‐target arthropods were assessed; (iv) the papers reported data on development, survival and reproduction as response variables for non‐target taxa in laboratory studies, and species abundances in field studies; (v) each study reported means accompanied by standard deviations (SD) or standard error (SE) and sample size (*n*); (vi) all studies were conducted in China and published in English or Chinese up to September 2016.

To build the database, we followed the formulation described by Marvier *et al*. (2007). For each study, we recorded authors and journal information, details about the *Bt* rice (Cry protein, transgenic event and its control), the non‐target group (taxonomy, functional guild and stage) and the experimental treatment with its control. We also recorded study location, cultivation, plot size, exposure method, sampling method and other methodological details. If a study reported figures without numerical data, we used ImageJ software version 1.45 to measure means and its variance (Abràmoff *et al*., 2004). We contacted authors to obtain details when needed.

We developed data eligibility criteria for laboratory and field studies. For laboratory studies, we used (i) total eggs per female for reproduction, immature stage survival rate, adult emergence and pupation rate, but not egg‐hatching data for survival; (ii) we selected the separately recorded male and female development time and the entire time span of larval or nymphal development; (iii) if studies reported multiple generation effects, we used the last generation for analyses. The criteria for field studies were (i) we chose the seasonal means and, when they were not available, the peak abundance; (ii) when multiple life‐stage data was reported in one study, we chose the larval or nymphal abundance; (iii) we used the ecological functional groups, not individual species. However, when analysing by taxonomic group, we used the individual species.

We identified the ecological functional guilds specified in the original papers. If a paper did not identify the guilds, we classified the non‐target taxa into five functional guilds: parasitoids, predators, herbivores, detritivores and others, following the description of Liu *et al*. (2003; Table S3).

### Data analysis

Before the quantitative data synthesis, publication bias of the database was conducted by a funnel plot with the Begg–Mazumdar rank correlation test and Egger's regression test (Begg and Mazumdar, 1994; Egger *et al*., 1997; Haworth *et al*., 2016). A symmetrical funnel plot showed no publication bias of the data (Field and Gillett, 2010). Trim‐and‐fill method was used to balance the asymmetrical funnel plot (Duval and Tweedie, 2000).

A weighted mean effect size (*E*), Hedges’*d*, was used to calculate the difference between an experimental (*Bt*) and the control (non‐*Bt*) mean divided by the pooled standard deviation and weighted by the reciprocal of the sampling variance (Hedges and Olkin, 1985). A negative effect size value indicates the *Bt* group had lower abundance, fecundity, survival or shorter development time compared with the non‐*Bt* group, and a positive value indicates these parameters were higher or longer than controls. For hypothesis testing, we used the parametric 95% confidence interval (*CI*) to test the results. If the interval enclosed zero, we took the effect size as not significantly different from zero. We assessed heterogeneity with the *Q* test and *I* ^2^ statistic. When the *I* ^2^ value was larger than 50% and *P*_heterogeneity_ < 0.001, we considered the data highly heterogeneous and conducted subgroup analysis to analyse the high heterogeneity. The random‐effect model was the more appropriate method to carry out all the analysis for the data included in the analysis came from different research groups (Borenstein *et al*., 2009; Schmidt *et al*., 2009). All the analyses were conducted using the STATA software version 12.0 (STATA Crop, College Station, TX).

## Conflict of interest

The authors declare no conflicts of interest.

## Supporting information


**Figure S1** Publication bias test for the laboratory data.
**Figure S2** Publication bias test for the field data.
**Figure S3** Trim‐and‐fill method to explain the publication bias of the abundance data of herbivores and parasitoids.
**Figure S4** Meta‐analysis of laboratory studies examining non‐target effects of transgenic *Bt* rice on herbivores reproduction.
**Figure S5** Meta‐analysis of laboratory studies examining non‐target effects of transgenic *Bt* rice on predators development.
**Figure S6** Meta‐analysis of laboratory studies and field studies examining non‐target effects of transgenic *Bt* rice on planthoppers.
**Table S1** Summary of meta‐database used in analysis of laboratory studies.
**Table S2** Summary of meta‐database used in analysis of field studies.
**Table S3** Functional guilds classification in analysis.
