# Supplementary material for: Does Bt rice pose risks to non‐target arthropods? Results of a meta‐analysis in China
Source: Plant Biotechnol J. 2017 Feb 20;15(8):1047–53. doi: 10.1111/pbi.12698 (PMC5506656; doi:10.1111/pbi.12698)
Supplement: Supplementary file 1 — Figure S1 Publication bias test for the laboratory data. Figure S2 Publication bias test for the field data. Figure S3 Trim‐and‐fill method to explain the publication bias of the abundance data of herbivores and parasitoids. Figure S4 Meta‐analysis of laboratory studies examining non‐target effects of transgenic Bt rice on herbivores reproduction. Figure S5 Meta‐analysis of laboratory studies examining non‐target effects of transgenic Bt rice on predators development. Figure S6 Meta‐analysis of laboratory studies and field studies examining non‐target effects of transgenic Bt rice on planthoppers. Table S1 Summary of meta‐database used in analysis of laboratory studies. Table S2 Summary of meta‐database used in analysis of field studies. Table S3 Functional guilds classification in analysis. [file PBI-15-1047-s001.docx]

**Supporting Information**

**Supplementary figure legends**

**Fig. S1.** Publication bias test for the laboratory data. For the reproduction of parasitoids, two cases were too limited to make the publication bias test. For the development of detritivores, no data could be collected to conduct the test.

**Fig. S2.** Publication bias test for the field data.

**Fig. S3.** The trim and fill method to explain the publication bias of the abundance data of herbivores and parasitoids. Data from studies used in meta-analysis is represented by solid gray circles, and the added studies after trim and fill method is represented by hollow circles.

**Fig. S4.** Meta-analysis of laboratory studies examining non-target effects of transgenic *Bt* rice on herbivores reproduction.

**Fig. S5.** Meta-analysis of laboratory studies examining non-target effects of transgenic *Bt* rice on predators development.

**Fig. S6.** Meta-analysis of laboratory studies and field studies examining non-target effects of transgenic *Bt* rice on planthoppers.


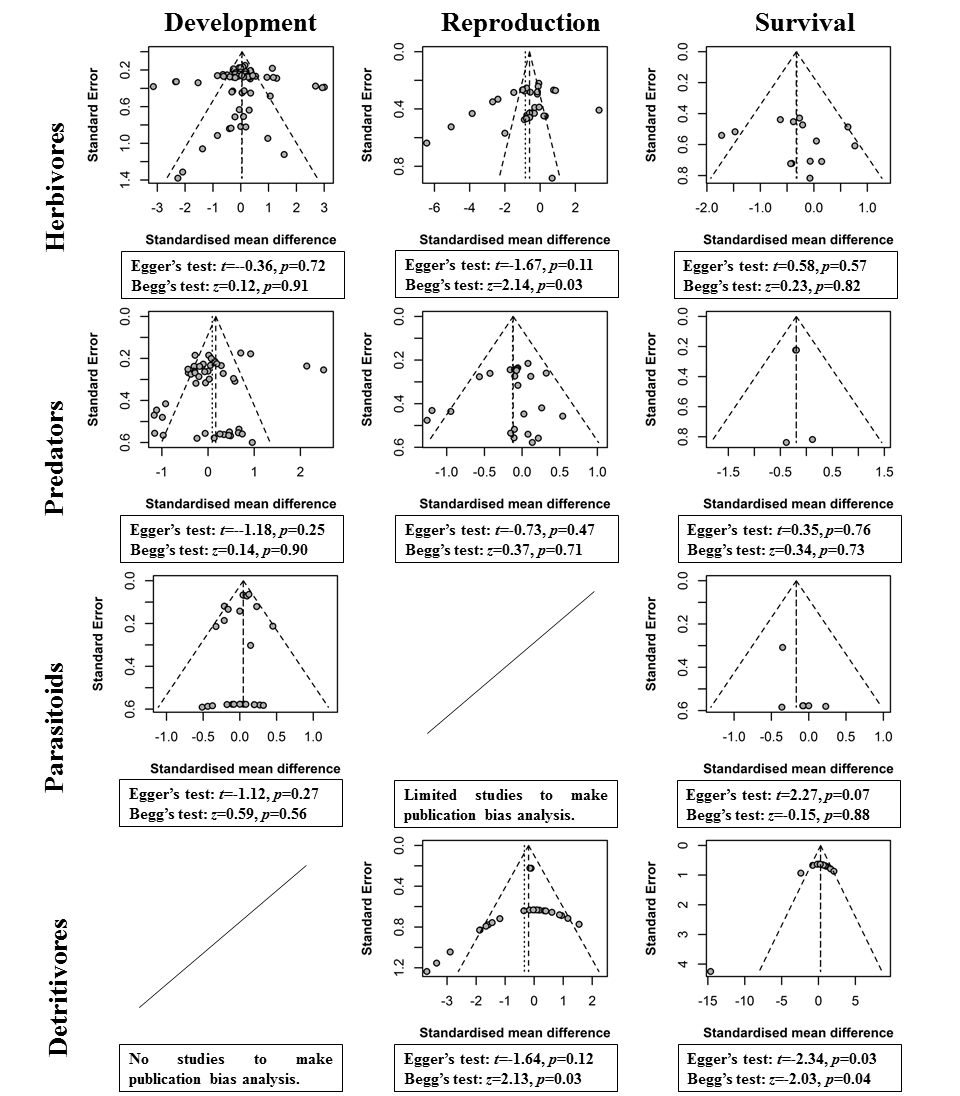


**Fig. S1.**

**
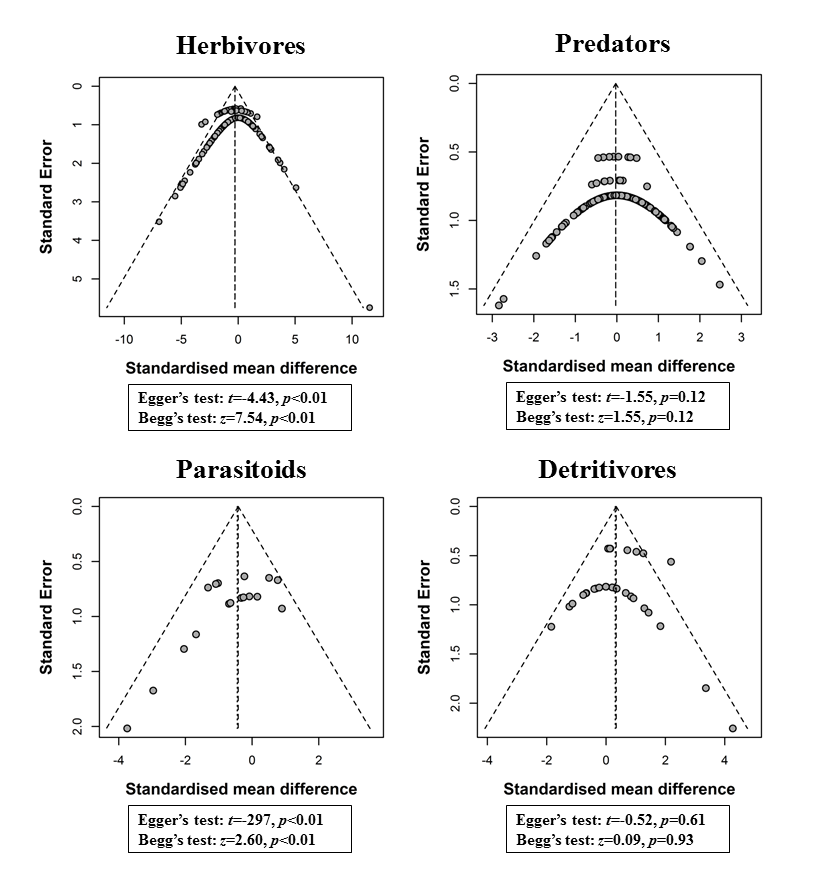
**

**Fig. S2.**


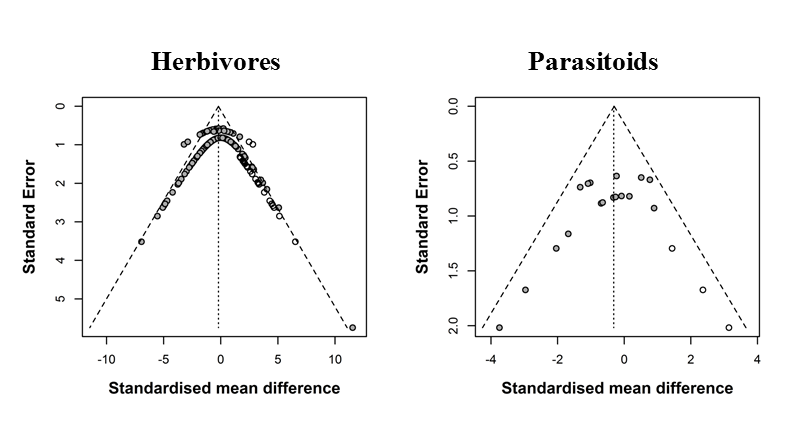


**Fig. S3.**



**Fig. S4.**



**Fig. S5.**

**

Fig. S6.**

**Supplementary tables**

**Table S1.** Summary of meta-database used in analysis of laboratory studies

| Functional guild | Cry protein | *Bt* event | Exposure method | Response variable for analyses | Number of  true replicates  (range) | Reference |
| --- | --- | --- | --- | --- | --- | --- |
| Herbivores | Cry1Ab | KMD2, KMD1, B1, B6 | *Bt* plant | survival, development, reproduction | 6-42 | [Akhtar *et al.* (2010](#_ENREF_1)); [Chen *et al.* (2012](#_ENREF_23)); Ren *et al*. (2016); [Tan *et al.* (2006](#_ENREF_75)) |
|  | Cry1Ab/Cry1Ac | TT51, TT9-3, TT9-4, not specified | *Bt* plant, *Bt* rice grain | survival, development, reproduction | 6-32 | [Akhtar *et al.* (2010](#_ENREF_1)); [Cai *et al.* (2008](#_ENREF_15)); [Mannakkara *et al.* (2013](#_ENREF_60)) |
|  | Cry1Ab/Vip3H+epsps | G6H1, G8-7 | *Bt* plant | development, reproduction | 30-40 | [Chen *et al.* (2011](#_ENREF_21)); [Lu *et al.* (2014a](#_ENREF_55)); Ren *et al*. (2016) |
|  | Cry1C | T1C-19 | *Bt* plant, *Bt* rice grain | development, reproduction, survival | 3-41 | Li *et al.* (2014a); [Liu *et al.* (2014](#_ENREF_50)); [Lu *et al.* (2014b](#_ENREF_57), d); [Mannakkara *et al.* (2013](#_ENREF_60)); [Wang *et al.* (2014](#_ENREF_81)a); [Zhang *et al.* (2011](#_ENREF_93)) |
|  | Cry2A | T2A-1 | *Bt* plant, *Bt* diet | development, reproduction, survival | 3-104 | Li *et al.* (2014a); [Liu *et al.* (2014](#_ENREF_50)); [Lu *et al.* (2014b](#_ENREF_57), d); [Mannakkara *et al.* (2013](#_ENREF_60)); [Wang *et al.* (2014](#_ENREF_81)b); [Zhang *et al.* (2011](#_ENREF_93)) |
|  | SCK + Cry1Ac | MSA, MSB | *Bt* plant | development, reproduction, survival | 6-68 | [Fu *et al.* (2003](#_ENREF_28)) |
| Predators | Cry1Ab | KMD1, KMD2 | *Bt* fed prey, *Bt* rice pollen, *Bt* diet | survival, development, reproduction | 3-58 | Akhtar *et al*. (2016); [Bai *et al.* (2005a](#_ENREF_5), b, [2006a](#_ENREF_8)); [Chen *et al.* (2009](#_ENREF_19)); [Li *et al.* (2014c](#_ENREF_46)); [Tian *et al.* (2010](#_ENREF_76), 2012); Zhou *et al*. (2016) |
|  | Cry1Ab/Cry1Ac | TT9-3, Huahui1 | *Bt* fed prey, *Bt* pollen, *Bt* diet | development, reproduction | 33-42 | [Tian *et al.* (2010](#_ENREF_77)); Zhou *et al*. (2016) |
|  | Cry1Ac | not specified | *Bt* diet | development | 30-37 | [Li *et al.* (2014c](#_ENREF_46)); Zhou *et al*. (2016) |
|  | Cry1C | T1C-19 | *Bt* rice pollen, *Bt* diet, *Bt* fed prey | survival, development, reproduction | 10-73 | [Li *et al.* (2014b](#_ENREF_44), 2015); Meng *et* *al*. (2016) |
|  | Cry2A | T2A-1 | *Bt* rice pollen, *Bt* diet, *Bt* fed prey | survival, development, reproduction | 11-73 | [Han *et al.* (2014, 2015a](#_ENREF_31)); [Li *et al.* (2013, 2014c](#_ENREF_46), 2015); [Wang *et al.* (2012](#_ENREF_82)) |
| Parasitoids | Cry1Ab | KMD1, KMD2 | *Bt* fed host,  *Bt* fed target host | development, survival | 6-514 | [Gao *et al.* (2010](#_ENREF_29)); [Jiang *et al.* (2004](#_ENREF_38)) |
|  | Cry2A | T2A-1 | *Bt* fed host,  *Bt* diet | development, survival | 12-780 | [Han *et al.* (2015b](#_ENREF_33)) |
|  | SCK + Cry1Ac | MSA, MSB | *Bt* fed target host | development, survival | 6 | [Jiang *et al.* (2005](#_ENREF_39)) |
| Detritivores | Cry1C | T1C-19 | *Bt* diet | survival, reproduction | 30-40 | [Yang *et al.* (2015](#_ENREF_87)) |
|  | Cry2A | T2A-1 | *Bt* diet | survival, reproduction | 30-40 | [Yang *et al.* (2015](#_ENREF_87)) |
|  | Cry1Ab | KMD1, KMD2 | *Bt* plant | survival, reproduction | 5 | [Bai *et al.* (2011](#_ENREF_10)); [Yuan *et al.* (2011](#_ENREF_90), 2013) |
|  | Cry1Ab/Cry1Ac | Huahui1, *Bt* Shanyou63 | *Bt* plant, *Bt* diet | survival, reproduction | 5 | [Yuan *et al.* (2011](#_ENREF_90), 2013) |

**Table S2.** Summary of meta-database used in analysis of field studies

| Functional guild | Cry protein | *Bt* event | Exposure method | Plot size (range, m^2^) | Number of true replicates  (range) | Year of experiment | Reference |
| --- | --- | --- | --- | --- | --- | --- | --- |
| Herbivores | Cry1Ab/Cry1Ac | TT9-3, TT9-4, TT51 | *Bt* plant | 500-700 | 3-7 | 2000, 2003, 2004, 2008, 2009, 2010 | [Akhtar *et al.* (2010](#_ENREF_1)); [Chen *et al.* (2006](#_ENREF_20)); [Han *et al.* (2011](#_ENREF_34)); [Liu *et al.* (2002](#_ENREF_51)) |
|  | Cry1C | T1C-19 | *Bt* plant | 225-500 | 3 | 2009, 2010, 2011, 2012 | [Han *et al.* (2011](#_ENREF_34)); [Lu *et al.* (2014b](#_ENREF_57), c, d) |
|  | Cry2A | T2A-1 | *Bt* plant | 225-500 | 3 | 2011, 2012 | [Han *et al.* (2011](#_ENREF_34)); [Lu *et al.* (2014b](#_ENREF_57), c, d) |
|  | Cry1Ab | KMD1, KMD2, B1, B6 | *Bt* plant, *Bt* rice residues | 225-700 | 3-6 | 2002, 2003, 2006, 2007, 2008, 2009, 2011, 2013 | [Akhtar *et al.* (2010](#_ENREF_1), 2013a, b); [Bai *et al.* (2012](#_ENREF_12)); [Chen *et al.* (2007](#_ENREF_17)); Ren *et al*. (2016) |
|  | Cry1Ab/Vip3H+epsps | G6H1, G8-7 | *Bt* plant | 150-700 | 3-5 | 2010, 2011, 2012, 2013 | [Lu *et al.* (2013, 2014a](#_ENREF_55), 2016); Ren *et al*. (2016) |
| Predators | Cry1Ab/Cry1Ac | TT9-3, TT9-4, TT51 | *Bt* plant | 333-500 | 3-7 | 2000, 2005, 2006, 2007, 2009, 2010 | [Han *et al.* (2011](#_ENREF_34)); [Liu *et al.* (2002](#_ENREF_51)); [Tian *et al.* (2010](#_ENREF_77)); [Xu *et al.* (2011](#_ENREF_85)) |
|  | Cry1C | T1C-19 | *Bt* plant | 225-500 | 3 | 2009, 2010, 2011, 2012 | [Han *et al.* (2011](#_ENREF_34)); [Lu *et al.* (2014c](#_ENREF_58)); [Xu *et al.* (2011](#_ENREF_85)) |
|  | Cry2A | T2A-1 | *Bt* plant | 150-500 | 3-4 | 2009, 2010, 2011, 2012, 2013 | [Han *et al.* (2011, 2014, 2015a](#_ENREF_31)); [Lu *et al.* (2014c](#_ENREF_58)); [Xu *et al.* (2011](#_ENREF_85)) |
|  | Cry1Ab | KMD1, KMD2, B1, B6 | *Bt* plant, *Bt* rice residues | 225-700 | 3 | 2002, 2003, 2004, 2005, 2006, 2007, 2009, 2011 | [Akhtar *et al.* (2013b](#_ENREF_3), [2016](#_ENREF_3)); [Bai *et al.* (2012](#_ENREF_12)); [Chen *et al.* (2007](#_ENREF_17), [2009](#_ENREF_19)); [Tian *et al.* (2010](#_ENREF_77)) |
|  | Cry1Ab/Vip3H+epsps | G6H1 | *Bt* plant | 700 | 3 | 2010, 2011, 2012 | Lu *et al.* (2016) |
|  | Cry1Ac+CpTI | II You Kefeng 6 | *Bt* plant | 333-667 | 3 | 2005, 2009 | Lin *et al.* (2016) |
| Parasitoids | Cry1C | T1C-19 | *Bt* plant | 225 | 3 | 2011, 2012 | [Lu *et al.* (2014c](#_ENREF_58)) |
|  | Cry2A | T2A-1 | *Bt* plant | 225 | 3 | 2011, 2012 | [Lu *et al.* (2014c](#_ENREF_58)) |
|  | Cry1Ab | KMD1, KMD2 | *Bt* rice residues, *Bt* plant | 315-500 | 3-5 | 2000, 2001, 2006, 2007 | [Bai *et al.* (2012](#_ENREF_12)); [Tian *et al.* (2008](#_ENREF_78)) |
|  | Cry1Ab/Vip3H+epsps | G6H1 | *Bt* plant | 700 | 3 | 2010, 2011, 2012 | Lu *et al.* (2016) |
| Detritivores | Cry1C | T1C-19 | *Bt* plant | 225 | 3 | 2011, 2012 | [Lu *et al.* (2014c](#_ENREF_58)) |
|  | Cry2A | T2A-1 | *Bt* plant | 225 | 3 | 2011, 2012 | [Lu *et al.* (2014c](#_ENREF_58)) |
|  | Cry1Ab | KMD1, KMD2, B1, B6 | *Bt* rice residues, *Bt* plant | 315-500, not specified | 3-12 | 2003, 2004, 2005, 2006, 2007, | [Bai *et al.* (2005c](#_ENREF_7), [2006b](#_ENREF_9), [2010](#_ENREF_11), [2012](#_ENREF_12)) |
|  | Cry1Ab/Vip3H+epsps | G6H1 | *Bt* plant | 700 | 3 | 2010, 2011, 2012 | Lu *et al.* (2016) |
| Others | Cry1C | T1C-19 | *Bt* plant | 225 | 3 | 2011, 2012 | [Lu *et al.* (2014c](#_ENREF_58)) |
|  | Cry2A | T2A-1 | *Bt* plant | 225 | 3 | 2011, 2012 | [Lu *et al.* (2014c](#_ENREF_58)) |
|  | Cry1Ab/Vip3H+epsps | G6H1 | *Bt* plant | 700 | 3 | 2010, 2011, 2012 | Lu *et al.* (2016) |

**Table S3.** Functional guilds classification in analysis

| Functional guilds | Orders | Family |
| --- | --- | --- |
| Herbivores | Hemiptera | Delphacidae, Cicadellidae, Aphididae, Miridae |
|  | Thysanoptera | Phlaeothripidae, Thripidae |
|  | Diptera | Ephydridae, Cecidomyiidae, Chloropidae |
|  | Coleoptera | Tenebrionidae |
| Predators | Hemiptera | Anthocoridae, Miridae, Veliidae |
|  | Araneae | Araneidae, Linyphiidae, Lycosidae, Micryphantidae, Tetragnathidae, Theridiidae |
|  | Neuroptera | Chrysopidae |
|  | Coleoptera | Coccinellidae, Staphylinidae |
|  | Diptera | Empididae |
| Parasitoids | Hymenoptera | Braconidae, Diapriidae, Eulophidae, Ichneumonidae, Mymaridae, Pteromalidae |
| Detritivores | Collembola | Entomobryidae, Hypogastruridae, Isotomidae, Sminthuridae |
|  | Diptera | Ceratopogonidae, Phoridae, Psychodidae |
| Others | Diptera | Culicidae, Drosophilidae |

**Supplementary references (references in Table S1-S2)**

Akhtar, Z.R., Dang, C., Peng, Y.F. and Ye, G.Y. (2016) Thrips-mediated impacts from transgenic rice expressing Cry1Ab on ecological fitness of non-target predator *Orius tantilus* (Hemiptera: Anthocoridae). *J. Integr. Agric*. **15**, 2059-2069.

Akhtar, Z.R., Tian, J.C., Chen, Y., Fang, Q., Hu, C., Chen, M., Peng, Y.F. and Ye, G.Y. (2010) Impacts of six *Bt* rice lines on nontarget rice feeding thrips under laboratory and field conditions. *Environ. Entomol*. **39**, 715-726.

Akhtar, Z.R., Tian, J.C., Chen, Y., Fang, Q., Hu, C., Peng, Y.F. and Ye, G.Y. (2013a) Impact of six transgenic *Bacillus thuringiensis* rice lines on four nontarget thrips species attacking rice panicles in the paddy field. *Environ. Entomol.* **42**, 173-180.

Akhtar, Z.R., Ye, G.Y., Lu, Z.B., Chang, X., Shen, X.J., Peng, Y.F. and Hu, C. (2013b) Impact assessments of transgenic *cry1Ab* rice on the population dynamics of five non-target thrips species and their general predatory flower bug in *Bt* and non-*Bt* rice fields using color sticky card traps. *J. Integr. Agric.* **12**, 1807-1815.

Bai, Y.Y., Jiang, M.X. and Cheng, J.A. (2005a) Effects of transgenic *cry1Ab* rice pollen on fitness of *Propylea japonica* (Thunberg). *J. Pest Sci.* **78**, 123-128.

Bai, Y.Y., Jiang, M.X. and Cheng, J.A. (2005b) Effects of transgenic *cry1Ab* rice pollen on the oviposition and adult longevity of *Chrysoperla sinica* Tjeder. *Acta Phytophy. Sin.* **32**, 225-230

Bai, Y.Y., Jiang, M.X. and Cheng, J.A. (2005c) Impacts of transgenic *cry1Ab* rice on two collembolan species and predation of *Microvelia horvathi* (Hemiptera: Veliidae). *Acta Entomol. Sin.* **48**, 42-47.

Bai, Y.Y., Jiang, M.X., Cheng, J.A. and Wang, D. (2006a) Effects of Cry1Ab toxin on *Propylea japonica* (Thunberg) (Coleoptera: Coccinellidae) through its prey, *Nilaparvata lugens* Stål (Homoptera: Delphacidae), feeding on transgenic *Bt* rice. *Environ. Entomol.* **35**, 1130-1136.

Bai, Y.Y., Jiang, M.X., Cheng, J.A. and Wang, D. (2006b) Effects of transgenic *Bt* *cry1Ab* rice on collembolan population in paddy field. *Chin. J. Appl. Ecol.* **17**, 903-906.

Bai, Y.Y., Yan, R.H., Ke, X., Ye, G.Y., Huang, F.N., Luo, Y.M. and Cheng, J.A. (2011) Effects of transgenic *Bt* rice on growth, reproduction, and superoxide dismutase activity of *Folsomia candida* (Collembola: Isotomidae) in laboratory studies. *J. Econ. Entomol.* **104**, 1892-1899.

Bai, Y.Y., Yan, R.H., Ye, G.Y., Huang, F.N. and Cheng, J.A. (2010) Effects of transgenic rice expressing *Bacillus thuringiensis* Cry1Ab Protein on ground-dwelling collembolan community in postharvest seasons. *Environ. Entomol.* **39**, 243-251.

Bai, Y.Y., Yan, R.H., Ye, G.Y., Huang, F.N., Wangila, D.S., Wang, J.J. and Cheng, J.A. (2012) Field response of aboveground non-target arthropod community to transgenic *Bt*-*Cry1Ab* rice plant residues in postharvest seasons. *Transgenic Res.* **21**, 1023-1032.

Cai, W.L., Zhang, H.Y., Yang, S., Yang, C.J., Hua, H.X. and Peng, Y.F. (2008) Impact of *Bt* rice grain on the development of red flour beetle, *Tribolium castaneum* (Coleoptera: Tenebrionidae). *Acta Phytophy. Sin.* **35**, 471-472.

Chen, M., Liu, Z.C., Ye, G.Y., Shen, Z.C., Hu, C., Peng, Y.F., Altosaar, I. and Shelton, A.M. (2007) Impacts of transgenic *cry1Ab* rice on non-target planthoppers and their main predator *Cyrtorhinus lividipennis* (Hemiptera: Miridae) - a case study of the compatibility of *Bt* rice with biological control. *Biol. Control*, **42**, 242-250.

Chen, M., Ye, G.Y., Liu, Z.C., Fang, Q., Hu, C., Peng, Y.F. and Shelton, A.M. (2009) Analysis of Cry1Ab toxin bioaccumulation in a food chain of *Bt* rice, an herbivore and a predator. *Ecotoxicology*, **18**, 230-238.

Chen, M., Ye, G.Y., Liu, Z.C., Yao, H.W., Chen, X.X., Shen, S.Z., Hu, C. and Datta, S.K. (2006) Field assessment of the effects of transgenic rice expressing a fused gene of *cry1Ab* and *cry1Ac* from *Bacillus thuringiensis* Berliner on nontarget planthopper and leafhopper populations. *Environ. Entomol.* **35**, 127-134.

Chen, Y., Tian, J.C., Peng, Y.F., Guo, Y.Y. and Ye, G.Y. (2011) Multi-generation effects of transgenic *cry1Ab/vip3H* rice G6H1 on development and reproduction of the non-target pest, *Nilaparvata lugens* (Stål). *Chin. J. Biol. Control*, **27**, 490-497.

Chen, Y., Tian, J.C., Wang, W., Fang, Q., Akhtar, Z.R., Peng, Y.F., Hu, C., Guo, Y.Y., Song, Q.S. and Ye, G.Y. (2012) *Bt* rice expressing Cry1Ab does not stimulate an outbreak of its non-target herbivore, *Nilaparvata lugens*. *Transgenic Res.* **21**, 279-291.

Fu, Q., Wang, F., Li, D.H., Yao, Q., Lai, F.X. and Zhang, Z.T. (2003) Effects of insect-resistant transgenic rice lines MSA and MSB on non-target pests *Nilaparvata lugens* and *Sogatella fucifera*. *Acta Entomol. Sin.* **46**, 697-704.

Gao, M.Q., Hou, S.P., Pu, D.Q., Shi, M., Ye, G.Y. and Chen, X.X. (2010) Multi-generation effects of *Bt* rice on *Anagrus nilaparvatae*, a parasitoid of the nontarget pest *Nilapavarta lugens*. *Environ. Entomol.* **39**, 2039-2044.

Han, Y., Chen, J., Wang, H., Zhao, J., He, Y.P. and Hua, H.X. (2015a) Prey-mediated effects of transgenic *cry2Aa* rice on the spider *Hylyphantes graminicola*, a generalist predator of *Nilapavarta lugens*. *Biocontrol*, **60**, 251-261.

Han, Y., Meng, J.R., Chen, J., Cai, W.L., Wang, Y., Zhao, J., He, Y.P., Feng, Y.N. and Hua, H.X. (2014) *Bt* rice expressing Cry2Aa does not harm *Cyrtorhinus lividipennis*, a main predator of the nontarget herbivore *Nilapavarta lugens*. *PLoS ONE*, **9**, e112315.

Han, Y., Wang, H., Chen, J., Cai, W.L. and Hua, H.X. (2015b) No impact of transgenic *cry2Aa* rice on *Anagrus nilaparvatae*, an egg parasitoid of *Nilaparvata lugens*, in laboratory tests. *Biol. Control*, **82**, 46-51.

Han, Y., Xu, X.L., Ma, W.H., Yuan, B.Q., Wang, H., Liu, F.Z., Wang, M.Q., Wu, G. and Hua, H.X. (2011) The influence of transgenic *cry1Ab/cry1Ac*, *cry1C* and *cry2A* rice on non-target planthoppers and their main predators under field conditions. *Agric. Sci. China*, **10**, 1739-1747.

Jiang, Y.H., Fu, Q., Cheng, J.A., Ye, G.Y., Bai, Y.Y. and Zhang, Z.T. (2004) Effects of transgenic *Bt* rice on the biological characteristics of *Apanteles chilonis* (Munakata) (Hymenoptera :Braconidae). *Acta Entomol. Sin.* **47**, 124-129.

Jiang, Y.H., Fu, Q., Cheng, J.A., Zhu, Z.R., Jiang, M.X. and Zhang, Z.T. (2005) Effect of transgenic *sck*+*cry1Ac* rice on the survival and growth of *Chilo suppressalis* (Walker) (Lepidoptera: Pyralidae) and its parasitoid *Apanteles chilonis* (Munakata) (Hymenoptera: Braconidae). *Acta Entomol. Sin.* **48**, 554-560.

Li, X.D., Yang, Y.J., Tian, J.C., Xu, H.X., Zheng, X.S. and Lü, Z.X. (2014a) Ecological responses of brown planthopper, *Nilaparvata lugens*, to triazophos and deltamethrin on transgenic *Bt* rice lines and their non-*Bt* parental rice. *Chin. J. Appl. Ecol.* **25**, 3006-3010.

Li, Y.H., Chen, X.P., Hu, L., Romeis, J. and Peng, Y.F. (2014b) *Bt* rice producing Cry1C protein does not have direct detrimental effects on the green lancewing *Chrysoperla sinica* (Tjeder). *Environ. Toxicol. Chem.* **33**, 1391-1397.

Li, Y.H., Hu, L., Romeis, J., Wang, Y.A., Han, L.Z., Chen, X.P. and Peng, Y.F. (2014c) Use of an artificial diet system to study the toxicity of gut-active insecticidal compounds on larvae of the green lacewing *Chrysoperla sinica*. *Biol. Control*, **69**, 45-51.

Li, Y.H., Wang, Y.Y., Romeis, J., Liu, Q.S., Lin, K.J., Chen, X.P. and Peng, Y.F. (2013) *Bt* rice expressing Cry2Aa does not cause direct detrimental effects on larvae of *Chrysoperla sinica*. *Ecotoxicology*, **22**, 1413-1421.

Li, Y.H., Zhang, X.J., Chen, X.P., Romeis, J., Yin, X.M. and Peng, Y.F. (2015) Consumption of *Bt* rice pollen containing Cry1C or Cry2A does not pose a risk to *Propylea japonica* (Thunberg) (Coleoptera: Coccinellidae). *Sci. Rep.* **5**, 7679.

Lin, S., Vasseur, L. and You, M.S. (2016) Seasonal variability in spider assemblages in traditional and transgenic rice fields. *Environ. Entomol.* **45**, 537-546.

Liu, K., Yang, Y.J., Tian, J.C., Xu, H.X., Zheng, X.S. and Lü, Z.X. (2014) Multi-generation effect of *Bt* rice with *cry1C* and *cry2A* on survival, development and reproduction of non-target pest *Sogatella furcifera* (Horvath). *Acta Agric. Zhejiangensis*, **26**, 730-735.

Liu, Z.C., Ye, G.Y., Hu, C. and Datta, S.K. (2002) Effects of *Bt* transgenic rice on population dynamics of main non-target insect pests and dominant spider species in rice paddies. *Acta Phytophy. Sin.* **29**, 138-144.

Lu, Z.B., Dang, C., Han, N.S., Shen, Z.C., Peng, Y.F., Stanley, D. and Ye, G.Y. (2016) The new transgenic *cry1Ab*/*vip3H* rice poses no unexpected ecological risks to arthropod communities in rice agroecosystems. *Environ. Entomol.* **45**, 518-525.

Lu, Z.B., Han, N.S., Tian, J.C., Peng, Y.F., Hu, C., Guo, Y.Y., Shen, Z.C. and Ye, G.Y. (2014a) Transgenic *cry1Ab/vip3H+epsps* rice with insect and herbicide resistance acted no adverse impacts on the population growth of a non-target herbivore, the white-backed planthopper, under laboratory and field conditions. *J. Integr. Agric.* **13**, 2678-2689.

Lu, Z.B., Han, N.S., Xu, G., Liu, Y.E., Hu, C., Peng, Y.F., Guo, Y.Y. and Ye, G.Y. (2013) Impacts of transgenic *cry1Ab/vip3H+epsps* japonica rice on the population dynamics of its non-target herbivores, rice leafhoppers, under field conditions. *Acta Entomol. Sin.* **56**, 1275-1285.

Lu, Z.B., Liu, Y.E., Han, N.S., Tian, J.C., Peng, Y.F., Hu, C., Guo, Y.Y. and Ye, G.Y. (2014b) Transgenic *cry1C* or *cry2A* rice has no adverse impacts on the life‐table parameters and population dynamics of the brown planthopper, *Nilaparvata lugens* (Hemiptera: Delphacidae). *Pest Manag. Sci.* **71**, 937-945.

Lu, Z.B., Tian, J.C., Han, N.S., Hu, C., Peng, Y.F., Stanley, D. and Ye, G.Y. (2014c) No direct effects of two transgenic *Bt* rice lines, T1C-19 and T2A-1, on the arthropod communities. *Environ. Entomol.* **43**, 1453-1463.

Lu, Z.B., Tian, J.C., Wang, W., Xu, H.X., Hu, C., Guo, Y.Y., Peng, Y.F. and Ye, G.Y. (2014d) Impacts of *Bt* rice expressing Cry1C or Cry2A protein on the performance of nontarget leafhopper, *Nephotettix cincticeps* (Hemiptera: Cicadellidae), under laboratory and field conditions. *Environ. Entomol.* **43**, 209-217.

Mannakkara, A., Niu, L., Ma, W.H. and Lei, C.L. (2013) Zero effect of *Bt* rice on expression of genes coding for digestion, detoxification and immune responses and developmental performances of brown planthopper *Nilaparvata lugens* (Stål). *J. Insect Physiol.* **59**, 985-993.

Meng, J.R., Mabubu, J.I., Han, Y., He, Y.P., Zhao, J., Hua, H.X., Feng, Y.N. and Wu, G. (2016) No impact of transgenic cry1C rice on the rove beetle *Paederus fuscipes*, a generalist predator of brown planthopper *Nilaparvata lugens*. *Sci. Rep.* **6**, 30303.

Ren, S.P., Yang, F., Gao, M.Q., Pu, D.Q., Shi, M., Ye, G.Y., Shen, Z.C. and Chen, X.X. (2016) Effects of transgenic *Bt* rice on nontarget *Rhopalosiphum maidis* (Homoptera: Aphididae). *Environ. Entomol*. **45**, 1090-1096.

Tan, H., Ye, G.Y., Shen, J.H., Peng, Y.F. and Hu, C. (2006) Effects of transgenic indica rice expressing a gene of *cry1Ab* with insect resistance on the development and reproduction of nontarget pest, *Sogatella furcifera* (Homoptera: Delphacidae). *Acta Phytophy. Sin.* **33**, 251-256.

Tian, J.C., Chen, Y., Li, Z.L., Li, K., Chen, M., Peng, Y.F., Hu, C., Shelton, A.M. and Ye, G.Y. (2012) Transgenic Cry1Ab rice does not impact ecological fitness and predation of a generalist spider. *PLoS ONE*, **7**, e35164.

Tian, J.C., Liu, Z.C., Chen, M., Chen, Y., Chen, X.X., Peng, Y.F., Hu, C. and Ye, G.Y. (2010) Laboratory and field assessments of prey-mediated effects of transgenic *Bt* rice on *Ummeliata insecticeps* (Araneida: Linyphiidae). *Environ. Entomol.* **39**, 1369-1377.

Tian, J.C., Liu, Z.C., Yao, H.W., Ye, G.Y. and Peng, Y.F. (2008) Impact of transgenic rice with a *cry1Ab* gene on parasitoid subcommunity structure and the dominant population dynamics of parasitoid wasps in rice paddy. *J. Environ. Entomol.* **30**, 1-7.

Wang, J.M., Ge, D.H., Chen, X.P., Ding, J.T. and Peng, Y.F. (2014a) Effect of transgenic *cry1C* rice on the development of the yellow mealworm (Coleoptera: Tenebrionidae). *Chin. J. Appl. Entomol*. **51**, 1190-1196.

Wang, Y.Y., Li, Y.H., Romeis, J., Chen, X.P., Zhang, J., Chen, H.Y. and Peng, Y.F. (2012) Consumption of *Bt* rice pollen expressing Cry2Aa does not cause adverse effects on adult *Chrysoperla sinica* Tjeder (Neuroptera: Chrysopidae). *Biol. Control*, **61**, 246-251.

Wang, Z. X., Lin, K. J., Romeis, J., Liu, Y. L., Liu, Z. W., Li, Y. H. and Peng, Y. F. (2014b). Use of a dietary exposure system for screening of insecticidal compounds for their toxicity to the planthopper *Laodelphax striatellus*. *Insect Sci*. **21**, 667-675.

Xu, X.L., Han, Y., Wu, G., Cai, W.L., Yuan, B.Q., Wang, H., Liu, F.Z., Wang, M.Q. and Hua, H.X. (2011) Field evaluation of effects of transgenic *cry1Ab*/*cry1Ac*, *cry1C* and *cry2A* rice on *Cnaphalocrocis medinalis* and its arthropod predators. *Sci. China Life Sci.* **54**, 1019-1028.

Yang, Y., Chen, X.P., Cheng, L.S., Cao, F.Q., Romeis, J., Li, Y.H. and Peng, Y.F. (2015) Toxicological and biochemical analyses demonstrate no toxic effect of Cry1C and Cry2A to *Folsomia candida*. *Sci. Rep.* **5**, 15619.

Yuan, Y., Ke, X., Chen, F., Krogh, P.H. and Ge, F. (2011) Decrease in catalase activity of *Folsomia candida* fed a *Bt* rice diet. *Environ. Pollut.* **159**, 3714-3720.

Yuan, Y.Y., Xiao, N.W., Krogh, P.H., Chen, F.J. and Ge, F. (2013) Laboratory assessment of the impacts of transgenic *Bt* rice on the ecological fitness of the soil non-target arthropod, *Folsomia candida* (Collembola: Isotomidae). *Transgenic Res.* **22**, 791-803.

Zhang, L., Lin, K.J., Li, F. and Hou, M.L. (2011) The biological effects of transgenic rice varieties with *cry1C* or *cry2A* on the non-target insect pest *Laodelphax striatellus*. *Plant Prot.* **37**, 120-125.

Zhou, X., Guo, Y.L., Kong, H., Zuo, J., Huang, Q.X., Jia, R.Z., Guo, A.P. and Xu, L. (2016) A comprehensive assessment of the effects of transgenic *cry1Ac*/*cry1Ab* rice Huahui 1 on adult *Micraspis discolor* (Fabricius) (Coleoptera: Coccinellidae). *PLoS ONE*, **11**, e0142714.
